# Carboniferous Onychophora from Montceau‐les‐Mines, France, and onychophoran terrestrialization

**DOI:** 10.1111/ivb.12130

**Published:** 2016-08-02

**Authors:** Russell J. Garwood, Gregory D. Edgecombe, Sylvain Charbonnier, Dominique Chabard, Daniel Sotty, Gonzalo Giribet

**Affiliations:** ^1^ School of Earth, Atmospheric and Environmental SciencesThe University of ManchesterManchesterM13 9PLUK; ^2^ Department of Earth SciencesThe Natural History MuseumLondonSW7 5BDUK; ^3^ Département Histoire de la TerreMuséum national d'Histoire naturelle, ParisF‐75005ParisFrance; ^4^ Muséum d'Histoire naturelle d'AutunF‐71400AutunFrance; ^5^ Museum of Comparative Zoology and Department of Organismic and Evolutionary BiologyHarvard UniversityCambridgeMassachusetts02138USA; ^6^ Department of Life SciencesThe Natural History MuseumLondonSW7 5BDUK

**Keywords:** terrestrialization, Onychophora, velvet worms, *Antennipatus montceauensis* gen. nov. sp. nov., Ecdysozoa, fossils, Carboniferous

## Abstract

The geological age of the onychophoran crown‐group, and when the group came onto land, have been sources of debate. Although stem‐group Onychophora have been identified from as early as the Cambrian, the sparse record of terrestrial taxa from before the Cretaceous is subject to contradictory interpretations. A Late Carboniferous species from the Mazon Creek biota of the USA,* Helenodora inopinata*, originally interpreted as a crown‐group onychophoran, has recently been allied to early Cambrian stem‐group taxa. Here we describe a fossil species from the Late Carboniferous Montceau‐les‐Mines Lagerstätte, France, informally referred to as an onychophoran for more than 30 years. The onychophoran affinities of *Antennipatus montceauensis* gen. nov., sp. nov. are indicated by the form of the trunk plicae and the shape and spacing of their papillae, details of antennal annuli, and the presence of putative slime papillae. The poor preservation of several key systematic characters for extant Onychophora, however, prohibits the precise placement of the Carboniferous fossil in the stem or crown of the two extant families, or the onychophoran stem‐group as a whole. Nevertheless, *A. montceauensis* is the most compelling candidate to date for a terrestrial Paleozoic onychophoran.

Velvet worms (phylum Onychophora) are among the most charismatic land invertebrates (e.g., Monge‐Nájera & Morera‐Brenes 2015), and the only strictly terrestrial animal phylum (Giribet, in Brusca et al. 2016). Despite long being associated with early Paleozoic lobopodians, such as the famous *Aysheaia pedunculata* Walcott 1911 from the Cambrian Burgess Shale, it has been questioned whether velvet worms are direct, albeit terrestrial, descendants of the morphologically varied and diverse Cambrian marine lobopodians (e.g., Ou et al. 2012). The latest phylogenetic analyses of lobopodians have strengthened the hypothesis that Onychophora are indeed nested within a grade of Cambrian lobopodians, one that includes many armored forms such as *Hallucigenia* spp. (Smith & Ortega‐Hernández 2014; Yang et al. 2015; Smith & Caron 2015). Although the onychophoran total group (i.e., all taxa more closely related to extant Onychophora than to Tardigrada or Euarthropoda) extends back to the early Cambrian, the fossil record of likely crown‐group onychophorans is sparse, and in our estimation is restricted to one species described from Late Cretaceous Burmite (Grimaldi et al. 2002). This fossil, *Cretoperipatus burmiticus* engel & grimaldi 2002, reveals some of the synapomorphies of the onychophoran crown‐group, including lobopods with spinous pads and claws. Purported onychophorans from the Miocene Dominican amber and Eocene Baltic amber (Poinar 1996, 2000) are unlikely to be Onychophora. Although both were described as velvet worms, the only known individual of *Tertiapatus dominicanus* poinar 2000 has arthropodized antennae and an articulated tergal exoskeleton, while that of *Succinipatopsis balticus* poinar 2000 has a surface with fine projections unlike onychophoran cuticle, and has no diagnostic characters of the group.

*Helenodora inopinata* thompson & jones 1980 was originally described from two specimens preserved in siderite concretions from the Middle Pennsylvanian Francis Creek Shale (Mazon Creek), in Illinois, USA. The species was interpreted as a crown‐group onychophoran (Thompson & Jones 1980). While poorly preserved terminal features make it difficult to distinguish this fossil from that of lobopodians in the onychophoran stem‐group, which are also known from this Lagerstätte, equivocal evidence for onychophoran‐like antennae and possible slime papillae was subsequently presented (Haug et al. 2012). These features and close affinities to extant Onychophora were, however, rejected in the most recent revision of *H. inopinata*, in which a phylogenetic analysis placed it in a region of the onychophoran stem‐group populated by Cambrian marine fossils (Murdock et al. 2016). Hence, the significance of this fossil species remains unclear; individuals of *H. inopinata* present onychophoran‐like trunk annulation (annuli, or plicae; non‐segmental tegumentary folds) and have ventrolateral appendages similar to lobopods. However, the consistent posterior orientation of the latter—if not associated with taphonomic processes—and the narrow plicae differ from modern onychophorans or from the Carboniferous fossils described herein.

A collection of better preserved fossils is known from the Stephanian Montceau‐les‐Mines, in France (Rolfe et al. 1982; Heyler & Poplin 1988; Pacaud et al. 1981; Perrier & Charbonnier 2014). These fossils, previously cited in the literature (e.g., Murienne et al. 2014), are yet to be properly characterized and described. Here we use traditional techniques, and the tools of virtual paleontology (Sutton et al. 2014), to study three specimens, providing a formal description of this species (Figs. 1, 2, 3). With well‐preserved, onychophoran‐like antennae (showing alternating long and short annuli, the distal ones becoming slightly wider; Fig. 4), likely slime papillae (Fig. 1I), annulated lobopods (Fig. 3B), and papillae on the trunk plicae (Fig. 1H), the species provides the most convincing evidence yet known for terrestrial Onychophora in the Carboniferous. Despite this, the few characters that allow the extant families Peripatidae and Peripatopsidae to be distinguished from each other (leakage of pigmentation in ethanol, the presence or absence of a diastema on the inner blades of the jaws, and the position of the genital opening) cannot be resolved in these fossils. Hence this species cannot be positioned more precisely as being part of the ingroup of either family (i.e., crown‐group Onychophora) or as the sister taxon to both (stem‐group Onychophora).

**Figure 1:**
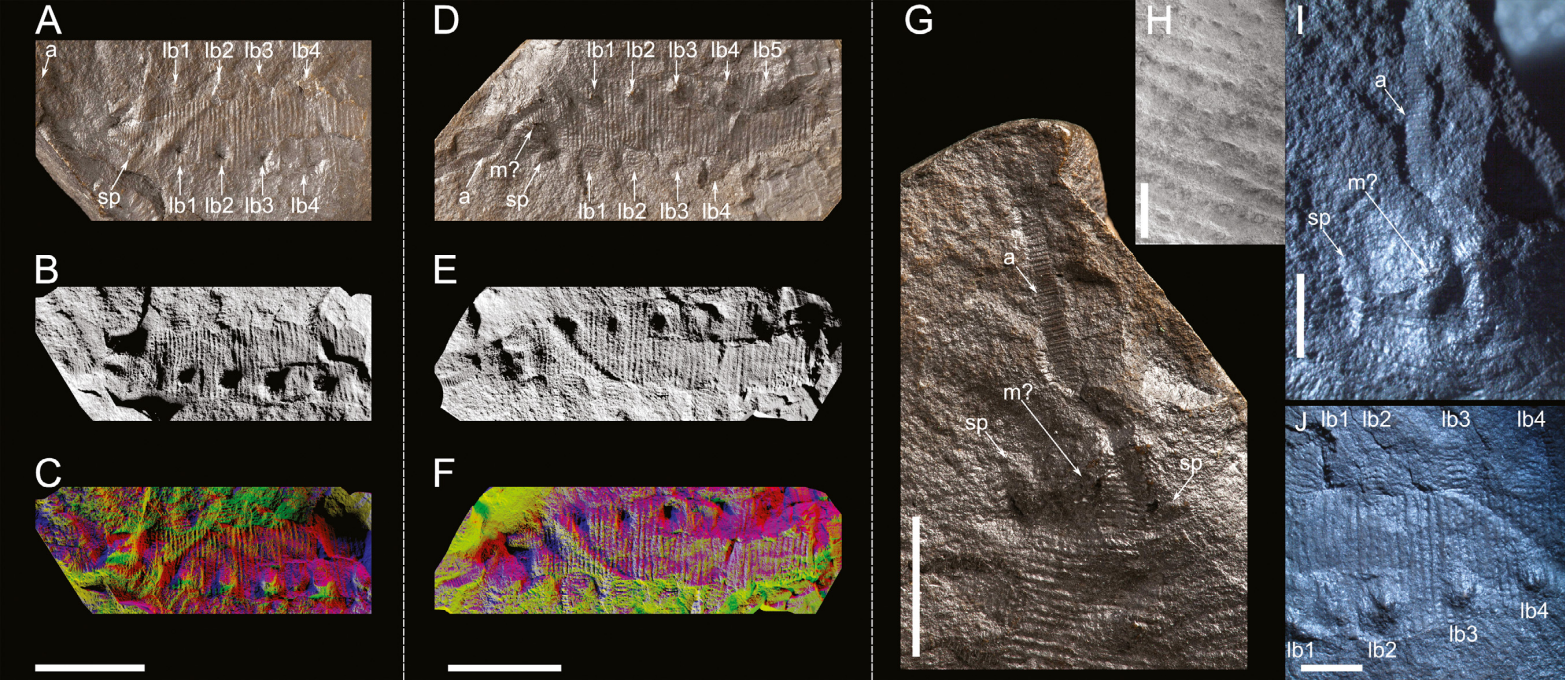
The Carboniferous onychophoran *Antennipatus montceauensis* gen. nov., sp. nov. from the Stephanian Montceau‐les‐Mines Lagerstätte, France. **A–C.** SOT003121a, showing the head, including an antenna and five anterior segments of the trunk. Shown in light photograph (A), as rendered image from CT data employing low‐angle lighting (B), and as a rendered image using a multicolored lighting rig (C). **D–E.** SOT003121b, showing the same region of the organism, but with one set of lobopods as protrusions rather than depressions. Shown as light photograph (D), low‐angle lighting render (E), and multicolored lighting render (F). **G.** An enlargement of 1D, showing the antenna, a possible mouth, and slime papilla. **H.** An SEM image of the trunk showing large primary papillae and ridges demarking plicae. **I.** A photomicrograph of the anterior of the fossil showing the left slime papilla, possible mouth, and antenna. **J.** A photomicrograph of the trunk, showing the plicae and several well‐preserved lobopods. a, antenna; lb1–lb5, lobopods 1–5; m?, putative mouth; sp, slime papilla. Scales: A–G, 10 mm; H, 1 mm; I–J, 2 mm.

**Figure 2:**
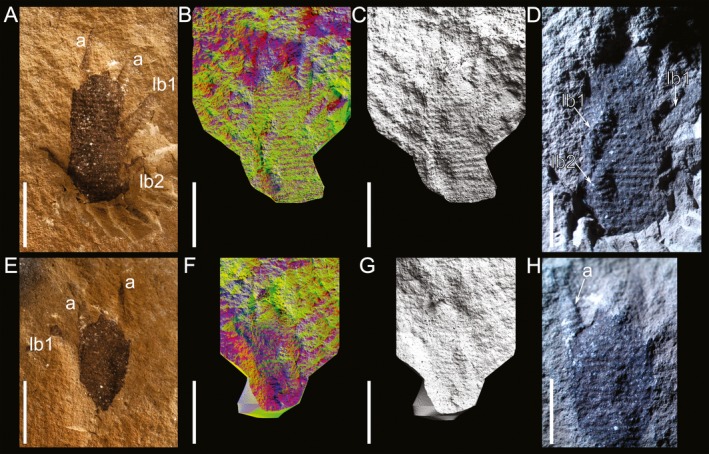
The Carboniferous onychophoran *Antennipatus montceauensis* gen. nov., sp. nov., specimen SOT006706a,b. **A–D.** SOT006706a, showing the head and anterior trunk of the organism, both antennae visible, and the anterior‐most lobopods. Posterior crack extended through preparation with a pen drill. Shown as light photograph (A), multicolored lighting render (B), and low‐angle lighting render (C). A photomicrograph (D) demonstrates both the demarcation of plicae through rows of papillae and the lobopods, which protrude on the right, but are folded over the body on the left. **E–H.** SOT006706b, showing the head and patchy preservation of the anterior‐most appendages. The antenna is better preserved on the right, but less clear than the body due to lack of dark coloration of the siderite. Light photograph (E), multicolored lighting render (F) and low‐angle lighting render (G). Photomicrograph (H) shows better preservation of the base of the left antenna, but this is incomplete. a, antenna; lb1 and lb2, lobopods 1 and 2. Scales: A–C, E–G, 5 mm; D and H, 2 mm.

**Figure 3:**
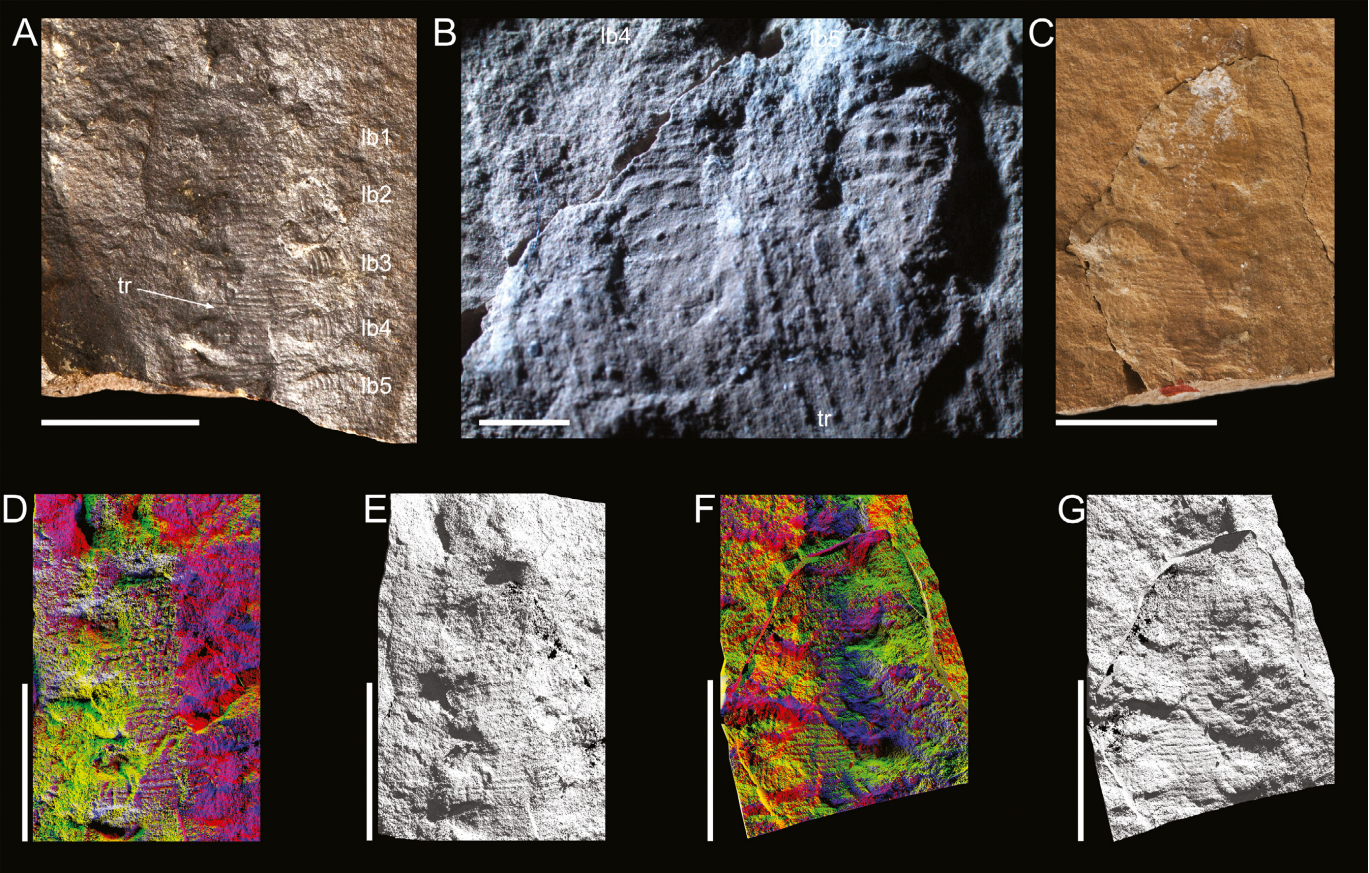
*Antennipatus montceauensis* gen. nov., sp. nov., specimen SOT003122a,b; the least well preserved of the three fossils. **A.** SOT003122b, most notable for the preservation of the plicae, by ridges and papillae, and details of the lobopods on the right. **B.** A photomicrograph of these lobopods, showing their attachment to the body, and segments demarcated by papillae and annuli. **C.** SOT003122a which is less well‐preserved, lacking the anterior‐most details through white mineral growth and cracking of the surface. **D–E.** SOT003122b showing through multicolored lighting render (D) and low‐angle lighting render (E). **F–G.** SOT003122a shown in multicolored lighting render (F) and low‐angle lighting render (G). lb1–lb4, lobopods 1–4; tr, trunk. Scales: A,C–G, 10 mm. B, 1 mm.

**Figure 4:**
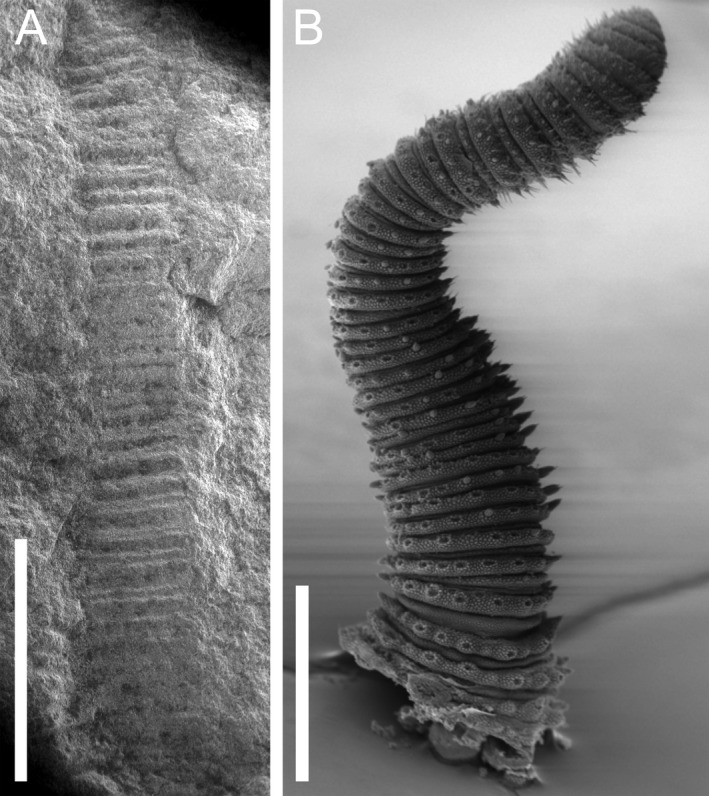
Comparative SEM images showing: **A.** The antenna of *Antennipatus montceauensis* gen. nov., sp. nov. (left), demonstrating alternating long and short annuli. **B.** The same pattern in an extant onychophoran species (*Epiperipatus isthmicola*; MNRJ 0092; Costa Rica; San Jose; courtesy of Cristiano Sampaio‐Costa). Scales: A, 2 mm. B, 0.5 mm.

## Methods

### Materials

We studied three fossils belonging to Collection Sotty 2, deposited in the Muséum d'Histoire naturelle d'Autun, but belonging to the Muséum national d'Histoire naturelle, Paris (MNHN). All are from the Montceau‐les‐Mines Lagerstätte, Assise de Montceau (Massif Central, France), and are Late Stephanian in age. These are specimen numbers SOT003121a,b (Fig. 1), SOT006706a,b (Fig. 2), and SOT003122a,b (Fig. 3). The fossils are found within siderite nodules, but lack the high level of three‐dimensional preservation seen in many other specimens from this site. They are instead low‐relief impressions revealed on the crack through which the nodule was split. All comprise part and counterpart. The specimens have been partially excavated with a pen drill.

### Photography and microscopy

Light photography was undertaken with a Canon EOS 5D Mark II camera, and with a Leica DCF290 camera attached to a Leica MZ16A microscope. Images stacks at set focal lengths were combined using the open source software ImageJ to reduce noise, and at different focal lengths using the freely available software CombineZM. Scanning electron microscopy of cuticular details used a FEI Quanta 650 FEG.

### CT scanning

The fossils were scanned on a Nikon HMX‐ST 225 scanner at The Natural History Museum, London, using a tungsten reflection target, 0.25 mm Cu filter, and 6284 projections at 708 ms exposure. Volumes were created using CT Pro 3D. The following parameters were employed: MNHN‐SOT003121—source current and voltage of 200 μA and 210 kV, reconstructed voxel size 20.4 μm; MNHN‐SOT003122—190 μA and 205 kV, 21.2 μm voxels; and MNHN‐SOT006706—190 μA and 205 kV, 18.9 μm voxels. Part and counterpart were scanned held together, allowing the crack to be visualized as outlined below. We note, however, that the recoverable surface in the scan of specimen MNHN‐SOT006706 was limited by the pen‐drill preparation which created excess void, obscuring details of the fossil.

### Visualization

We created digital visualizations by first thresholding and surfacing data with the SPIERS software suite (www.spiers-software.org), following the methods of Garwood & Sutton (2012). The resulting models were exported as VAXML datasets (Sutton et al. 2012) (Supporting information Appendix S1). Subsequently, STLs from these were imported into the open source raytracer Blender (www.blender.org) following the methods of Garwood & Dunlop (2014). Due to the low‐relief nature of the fossils, we present two different lighting schemes to highlight surface topography. Both employ Blender internal renderer. The first uses a single constant falloff spotlight at a low angle to the surface, with a low‐powered hemi light located close to the camera to lighten shadows. The other uses blue, red, green, and yellow spotlights at 90° to each other. For videos this lighting rig was rendered rotating around the fossil (Supporting information Video S1).

## Results

Phylum Onychophora Grube 1853

***Antennipatus*** **gen. nov.** garwood, edgecombe & giribet

### Etymology

The name refers to the onychophoran‐like antennae, which are well‐preserved enough in one of the specimens to place this fossil as a close relative to, or as a member of, crown‐group Onychophora.

### Diagnosis

Vermiform taxon with annulated paired appendages resembling lobopods (maximum length is 2.8 mm), serially repeated along a body covered by an annulated cuticle; each segment with eight plicae; each plica with papillae, their size and spacing similar to onychophoran primary papillae. Head with long onychophoran‐like antennae (with alternation of wide and narrow annuli) and possible slime papillae represented by ventrolateral appendages shorter than the other appendages. Length and total number of trunk segments not available; a maximum of five trunk segments preserved. (Claws and spinous pads of legs not observed; jaws not observed; genital opening and anal pore not preserved).

### Remarks

Morphological characters such as dermal papillae, foot papillae, and spinous pads, or the male anal gland openings (Oliveira et al. 2010), are traditionally used to define onychophoran genera. Because these are internal, or are features with low preservation potential, they cannot be observed, even in well‐preserved fossils such as those reported herein. They are hence of limited utility in the study of fossils. Furthermore, the phylogenetic significance of these characters has recently been challenged. While these characters have been used to define genera such as *Peripatus*,* Macroperipatus*, and *Epiperipatus*, all these taxa are non‐monophyletic in molecular phylogenetic analyses (Murienne et al. 2014; Oliveira et al. 2012a, 2013; C. Sampaio‐Costa, unpubl. data. 2016), and thus the characters are unreliable. In addition, the dated phylogeny of Murienne et al. (2014) suggests that our fossil predates the crown diversification of Peripatopsidae by more than 100 Ma, and overlaps with the error bar of the early diversification of Peripatidae. The only extant genus established by then is the southeast Asian peripatid *Eoperipatus*; because a single specimen was analyzed by Murienne et al. (2014), the time of *Eoperipatus* diversification remains uncertain. However, comparison to other taxa in the same study suggests that the crown‐group radiation of the genus was likely much more recent. It is thus unlikely that our fossil belongs to any extant onychophoran genus. In addition, given the high degree of endemicity in modern onychophoran genera (e.g., *Mesoperipatus* to the Bight of Biafra in West Africa, and the Neotropics host a series of mostly ill‐defined genera), and the fact that no extant Onychophora are known from Europe, we assign the new species to a new genus, *Antennipatus* gen. nov.

We note that a specimen, in ventral view, preserving the posterior end and with ten trunk segments, was illustrated as an onychophoran by Heyler & Poplin (1988). This has since been redescribed as a fireworm by Pleijel et al. (2004).

***Antennipatus montceauensis*** **gen. nov., sp. nov.** garwood, edgecombe, & giribet

### Synonymy

Poplin & Heyler (1994): “onychophore dans un nodule,” p.121. Perrier & Charbonnier (2014): “Undescribed onychophoran MNHN.F.SOT003121b,” p. 358, fig. 4d. Giribet *in* Brusca et al. (2016): “undescribed fossil Onychophora,” p. 719, fig. 20.11 g.

### Zoobank

Publication: urn:lsid:zoobank.org:pub:F8587BE6‐3475‐4B20‐9E60‐BD802F29906B

Genus: urn:lsid:zoobank.org:act:B98FC135‐0627‐43D5‐AA2C‐F9FB239994EA

Species: urn:lsid:zoobank.org:act:62525EC0‐5B81‐4A3B‐8BF7‐D9005D480041

### Etymology

The specific epithet refers to the origin of the fossil.

### Diagnosis

As for the genus.

### Type material

Holotype SOT003121a,b (part and counterpart); paratypes SOT003122a,b, and SOT006706a,b.

### Description, SOT003121

The more complete part of this specimen preserves a 7‐mm extent of one antenna and 26.5 mm encompassing the head and five segments of the trunk (Fig. 1). The sixth segment is incomplete (five plicae are preserved). The fossil is relatively flat, albeit with the lobopods of one side retaining limited 3‐D morphology and being directed into SOT003121a (Fig. 1A–C, thus preserved as protrusions in the surface of SOT003121b, Fig. 1D–F). The antenna is slender and gently tapers distally. There are in excess of 40 annuli of varying widths preserved on the antenna (Fig. 1G,I), with alternating longer and shorter annuli (Fig. 4), and annulus size increasing distally**.** It is incomplete distally, but its proximal attachment to the head is visible in SOT003121a. Immediately posterior to this is an elongate depression in this half of the nodule, corresponding to a protuberance, rounded in profile, in SOT003121b. This is longer anteroposteriorly (3.2 mm) than it is wide laterally (1.6 mm): this shape, coupled with the position ventral to the antennae and body margins at the anterior of the animal, is suggestive of a mouth (Fig. 1G,I). The visible structure could represent lip papillae (it is likely these would obscure the jaws in life), or sediment infill of the space between the lip papillae. To the left, aligned with the posterior of the putative mouth, is a 2.5‐mm lateral projection visible due to a small ridge outlined with depressions (Fig. 1I). The lower posterior portion of this ridge has been damaged by preparation with a pen drill, artificially narrowing its attachment to the body. Aligned with this on the right side of the body is a similar projection, in contrast to that on the other side, laterally directed, and 1.0 mm in length, which suggests that the structure was likely prematurely truncated or preserved withdrawn into the body. Both projections lack annulation, are shorter and narrower than the subsequent lobopods, taper, and have an anterolateral orientation. Based on the position, orientation, lack of obvious annulation (visible on all the lobopods posterior to this on this side of the fossil), we interpret these as slime papillae. Posterior to this is the trunk, 6.8 mm in width, with five lobopods, best preserved on the left side of SOT003121b (Fig. 1J). These are spaced ~3.4 mm, with eight plicae between each. Plicae are demarcated by lines of papillae (in excess of 20 across the preserved width of the animal per plica, all of a similar size, 180–250 μm diameter; detail of primary papillae is shown in Fig. 1H) and clear annulations in the form of transverse ridges. Maximum lobopod length is 2.8 mm, and lobopods show eight to ten annuli, each demarcated by papillae (varied in both diameter and relief), and in most cases annuli, in the form of depressions.

### Description, SOT006706

This specimen shows the head and anterior‐most part of the trunk (Fig. 2). The fossil is preserved in high relief, and much of the preserved portion of the animal is apparent as a dark residue in the siderite nodule, which records the cuticle surface with high fidelity. In some areas the dark residue is not present, but limited anatomy can still be observed through the relief of the crack through the nodule. The 12.6 mm of the fossil visible in SOT006706a is located within a depression in the crack (Fig. 2A–D). The faint impression of the antenna on the left corresponds to the well‐preserved, complete antenna on the right of the other part of the nodule, described below. The other antenna is better seen in this part; 4.3 mm of the distally tapering appendage are preserved, annuli are visible in some regions with ~200 μm spacing in these regions, but both long and short annuli are visible proximally. The presence of slime papillae is equivocal. It is possible one is preserved as a 1.5 mm anteriorly pointing protrusion on the left (Fig. 2A), demarcated from the body by a small crack. On the right, one could be represented by a shallow pit anterior to the first lobopod. The nature of the median anteriorly projecting dark patch is also equivocal. The preserved portion of the trunk of the specimen measures 10.2 mm in length, and 5.8 mm in width, with plicae demarcated by both ridges and lines of similarly sized papillae. There are eight plicae between each subsequent lobopod (Fig. 2D). The lobopods on this half of the nodule are directed toward the right. On the right a single lobopod is present, laterally projecting from the margin, 2.8 mm in length. Annulations and papillae are both visible. Legs posterior to this are either buried in matrix or obscured by preparation with a pen drill. Two left lobopods are visible in high relief, folded over on top of the trunk cuticle (Fig. 2C,D), with limited annuli and papillae visible on the posterior of the two. No further lobopods are visible, but the left margin of the body is very clear as result, with clear undulations for each plica. SOT006706b (Fig. 2E–H) preserves 7.4 mm of the anterior of the animal. Annulations are as reported for SOT006706a, and limbs preserved in positive relief on that half of the nodule are in negative relief here. The antenna on the right is almost complete (Fig. 2E) and is 4.5 mm in length, distally tapering. Morphology is clear due to altered color in the rock, but annulations are less apparent. Only the proximal 2.5 mm of the left is visible (Fig. 2H); the rest is seen in the counterpart. The slime papilla on the right is apparent as an anteriorly directed dark protrusion corresponding to that on the left of the other part. The left slime papilla is not clear in SOT006706b. The tip of the first left lobopod is preserved, continuing from the termination of the other part and disappears into the matrix.

### Description, SOT003122

This specimen is anteriorly the least well‐preserved of the three fossils (Fig. 3). The surface of SOT003122a is obscured by white mineral growth around the anterior of the animal, and the entire surface has chipped off and been glued back to leave a crack running around the fossil (Fig. 3C,F,G). This precludes reporting details of the head—all that is preserved is a small portion (<5 annuli) of a single antenna—but this part preserves details of the trunk annulation and lobopods. While 19.4 mm of the trunk length is preserved, the lateral margins are not clear, preventing estimates of the true width. The plicae are visible through both transverse ridges and lines of similarly sized papillae (some in this specimen are clearly lying along a ridge). There are eight plicae between each lobopod. No lobopods are complete, but proximal portions are present and demonstrate clear annulation through prominent papillae and ridges—the maximum preserved extent of tany limb is 3 mm, and there is a maximum of five annuli. Five lobopods are preserved. In SOT003122b (Fig. 3A,B,D,E), the proximal seven annuli of the right antenna are visible. The left is obscured by preparation artifacts from a pen drill, although level with the right antenna there are some possible annuli which may represent the proximal portion of the left antenna. Posterior to this is an anteroposteriorly elongate protuberance, rounded in profile, 1.8 mm×1.2 mm. This is in a position which is suggestive of a mouth. Posterior to this, 20.3 mm of trunk is preserved, with a maximum preserved width of 8.1 mm. Plicae are clearly demarcated by ridges (Fig. 3A,B) and lines of papillae with little size variation (a minimum of 12 are visible in the widest preserved region, with a spacing of <0.2 mm). There are eight plicae between each subsequent lobopod. Slime papillae and the anterior lobopods are not clearly preserved. Five lobopods are preserved as protuberances on the left and depressions on the right (Fig. 3B). The latter provide more information and increase in fidelity posteriorly, until the fossil is truncated by a terminal crack of the nodule. The best preserved lobopod (the fourth visible on the right) is 5.3 mm in length and has nine annuli preserved, which are demarcated by papillae and depressions or ridges. The lobopods’ terminations are not clearly preserved.

### Morphological remarks

The fossils of *A. montceauensis* gen. nov., sp. nov. reveal an organism with anteriorly directed, tapering onychophoran‐like antennae, with in excess of 40 annuli. The animal has an anteroposteriorly elongate mouth ventral to the antennal attachment. Just posterior to the antennae are tapering lateral slime papillae, lacking apparent annulation and shorter than subsequent lobopods (Fig. 1I). No eyes are apparent, although this is likely a combination of the preservation, where little endogenous material remains, and because even in life, onychophoran eyes can be morphologically subtle or even absent in a number of troglobitic species. The trunk has a minimum of five segments, each of which has eight plicae demarcated by lines of papillae and transverse ridges marking annulations (Fig. 1H). Tracheal openings are not apparent but are, like eyes, subtle features in extant taxa and would be difficult to observe in any non‐amber fossils. Lobopods are spaced consistent with a segmental nature, with eight plicae in each segment, demarcated by lines of papillae and depressions; terminal features such as claws are not preserved. The posterior of the animal, and thus the total number of body segments, and position of the anus and genital opening, remain unknown. Five are preserved in the 26.5 mm extent of SOT003121. An extrapolation based on extant onychophorans, which have between 19 and 43 leg pairs, would suggest individuals of the species were 10–20 cm long in life, a size reached by several extant Neotropical Peripatidae (Morera‐Brenes & Monge‐Nájera 2010).

The three specimens from Montceau‐les‐Mines reported herein are all identified as the same species based on the spacing, size, and shape of the plicae and papillae. This is also substantiated by the general pattern of finding a single species of onychophoran in most modern sites, although this need not be the case for the Carboniferous.

### Taxonomic remarks

Murdock et al. (2016) reviewed the history of nomenclature involving the names *Ilyodes divisa* Scudder 1890 and *Helenodora inopinata*, concluding that the former is based on a nomen dubium. The authors reinstated the latter—which had been placed in subjective synonymy with *I. divisa* by Pacaud et al. (1981; also Hay & Kruty 1997)—for the Carboniferous species from Mazon Creek that is most relevant to *A. montceauensis* (figured by Thompson & Jones 1980; Haug et al. 2012; Murdock et al. 2016). In *Helonodora inopinata*, there are nine plicae per segment in the trunk, whereas in *A. montceauensis,* there are eight. The two species also differ in the annulation and length of their lobopods relative to their body width; in *H. inopinata* these are short and stubby, being 2–3 mm in length relative to a body 6 mm wide (maximum width 13 mm fide Murdock et al. 2016). By contrast, in *A. montceauensis* the body is 8 mm wide but with significantly longer limbs, which are a minimum of 5.3 mm in length. The specimens of *A. montceauensis* have a minimum preserved length of 26.5 mm for a minimum of five segments, while the *H. inopinata* type specimen is 56 mm for the 20‐ (Murdock et al. 2016) to putatively 23‐segments (Thompson & Jones 1980). Hence, the Montceau species is significantly larger than that from Mazon Creek, for which a maximum length of 66 mm is reported (Murdock et al. 2016). However, we are reticent to assign any taxonomic significance to this observation because the difference could be ontogenetic. None of the published specimens of *H. inopinata* show annulation on the lobopods, whereas annulation is pronounced in the specimens of *A. montceauensis*. We initially considered this to be potentially taphonomic because the trunk in *H. inopinata* appears to be likewise largely non‐annulated. This is most likely due to preservation, because annulation is restricted to particular, irregular regions. Murdock et al. (2016), however, argue from decay experiments that the lack of annulation on the limbs in *H. inopinata* is primary. Where the trunk annulation is seen (Thompson & Jones 1980: Plate 2, fig. 3), the definition of the plicae and the size and arrangement of the papillae is comparable to that in *A. montceauensis*. The identity of putative papillae in *H. inopinata* has been called into question because similar structures are observed in unexpected positions, even external to the fossils (Murdock et al. 2016). In the case of *A. montceauensis*, however, we identify papillae on the trunk, and antennal and lobopod annuli with confidence.

## Discussion

### Affinities

Modern onychophorans divide into two families with non‐overlapping distributions: Peripatopsidae, with an Austral distribution (in the territories of the former temperate Gondwana: Australia, New Zealand, South Africa and Chile); and Peripatidae, with a few species in southeast Asia, one in west Africa, and its largest diversity in the Neotropical region (Bouvier 1905; Ruhberg 1985; Monge‐Nájera 1995; Oliveira et al. 2012b; Murienne et al. 2014). Molecular dating suggests that the two families diverged around the Devonian (Murienne et al. 2014), coincident with the origin of the oldest extensive forests. At this time, tree‐fern‐like taxa probably produced abundant litter, providing the potential for significant terrestrial carbon accumulation, and hence detritus‐based arthropod faunas (Stein et al. 2007). Extant onychophorans and many other typical leaf‐litter inhabitants are commonly found in tree‐fern litter in New Zealand. It is difficult to assign the fossil taxon to either family based on diagnostic morphological characters because the two families are distinguished by the position of the genital opening (between the penultimate leg pair in Peripatidae; between the last pair in Peripatopsidae), or by details of the inner blades of the jaws (with a diastema in Peripatidae, without it in Peripatopsidae; Mayer 2007). An additional character, the solubility of body pigments in ethanol, is irrelevant in the fossils. Therefore, due to a lack of these diagnostic characters, we cannot provide evidence in support of competing paleogeographic hypotheses. Considering the large body size of the fossil specimens, as well as a geographic distribution in the northern hemisphere, membership to the extant family Peripatidae might be expected, but cannot be demonstrated. It should be noted that the number of plicae per segment (eight in *Antennipatus montceauensis*) does not match that of extant Peripatidae, which is usually 12 or exceptionally 24 (Oliveira et al. 2014). The split between extant Peripatidae has been molecularly dated to the Permian (Murienne et al. 2014), so a Late Carboniferous fossil being a member of crown‐group Onychophora, or more specifically Peripatidae (either stem‐ or crown‐), would not be unexpected, given the likelihood of a Devonian split from Peripatopsidae (Murienne et al. 2014).

Phylogenetic analyses of Cambrian lobopodians and other panarthropods by Yang et al. (2015) and Murdock et al. (2016) encompassed three extant Onychophora (including exemplars of both extant families). These were collapsed in a polytomy with two fossil taxa, *Tertiapatus dominicanus*, from Dominican amber and *Helenodora inopinata* from Mazon Creek. As noted above, the status of *T. dominicanus* as an onychophoran is not well supported, and we regard it as likely to be an arthropod, rendering its positioning in Onychophora spurious. The lack of resolution was not caused by character conflict (causing branches to collapse in a consensus) but rather to a lack of informative characters for splitting either of the fossils from the crown‐group and assigning them to the stem‐group. The crux of the problem is that for likely autapomorphies of crown‐group Onychophora (such as a differentiated foot, or spinous pads on the lobopods) the fossils are coded as missing data. Therefore, they are free to float to any position in the onychophoran crown‐group. The same pertains to *A. montceauensis*, based on available data.

### Terrestrialization: evidence from morphology and depositional environment

*Antennipatus montceauensis* is among the earliest putative representatives of Onychophora, a phylum which is wholly terrestrial based on extant species. One of our fossils appears to have slime papillae (Fig. 1H). In the other specimen, with a well‐preserved head, slime papillae are equivocal (Fig. 2). Because these structures can be drawn into the body, and thus appear as little more than lateral swellings (Haug et al. 2012; Fig. S2F), their absence in one specimen does not contradict the hypothesis that they were present in the species as a whole. If this suggestion is correct, slime papillae would imply a terrestrial habitat: terrestrial onychophorans use a unique oscillation mechanism for shooting glue (Concha et al. 2015) that would not function in an aqueous environment. The observation would hence be in keeping with our posited placement of this species: a terrestrial onychophoran either in the crown‐group or in the terrestrialized part of the stem‐group. Other unequivocal morphological characters which would demonstrate terrestriality—beyond slime papillae—are unlikely to be resolved or preserved in fossils; for example, openings to the tracheal system are concentrated into atria, structures significantly smaller than 100 μm in size (Oliveira et al. 2012a).

Fossils of *Helenodora inopinata* are found associated with fossils of *Carbotubulus waloszeki* Haug et al. 2012; a long‐legged morphotype which is otherwise only known from Cambrian marine taxa (Haug et al. 2012), the functional morphology of whose limbs is also suggestive of a marine environment. Furthermore, the sedimentary environment of Pit 11 and the surrounding area, in which both were found, displays considerable marine influence (Baird et al. 1986). This is coupled with incomplete preservation, limiting speculation as to the mode of life of this Carboniferous species (Murdock et al. 2016). In contrast, the depositional environment of the Montceau‐les‐Mines Lagerstätte is freshwater, with significant terrestrial input. The host sediments represent a lacustrine/deltaic complex; varied paludal‐to‐fluvial environments are juxtaposed in the basin through synsedimentary faulting. For example, organic‐rich deposits are typically interrupted by detrital inputs of fluvial and lacustrine origin. There is no sedimentological, structural, or paleogeographic evidence for a marine influence (Racheboeuf et al. 2002, 2004, 2008; Perrier et al. 2006; Perrier & Charbonnier 2014). Based on available data, the closest Upper Carboniferous marine deposits were located at least several hundred kilometers to the southwest of Montceau (Courel et al. 1994). Evidence from the depositional environment of the sediments is reflected by the flora and fauna: tree trunks are preserved *in situ*, as are root layers, suggesting the sediments were deposited close to land (Charbonnier et al. 2008). A rich and diverse flora is represented by abundant compressions of lycopsids, sphenopsids, ferns, pteridosperms, and cordaites (Charbonnier et al. 2008; Perrier & Charbonnier 2014). Hygrophytic terrestrial plants flourished along the banks of Montceau water bodies, but more mesoxerophilic plants growing on the hills of the intermontane basin are also present as fossils (Langiaux 1994). The fauna of Montceau‐les‐Mines includes unequivocally terrestrial animals such as harvestmen (Garwood et al. 2011, 2014), trigonotarbids (Dunlop 1999), a spider (Selden 1996), myriapods (Racheboeuf et al. 2004), and insects (Garwood et al. 2012), as well as characteristically freshwater bivalves (Perrier & Charbonnier 2014). Thus, the depositional setting precludes the interpretation of *A. montceauensis* as a marine lobopodian. It is entirely plausible that it was a terrestrial organism, and if it were not, it must represent a freshwater, stem‐group onychophoran. Both scenarios place this fossil at a key point in onychophoran evolution.

## Author contributions

RG, GDE, and GG studied and described the specimens and RG, GDE, SC, and GG wrote the manuscript. DS collected the 100,000 nodules of the Montceau Lagerstätte, including these fossils. DC facilitated study of the specimens.

## Supporting information


**Appendix S1.** A zip file containing 3D models of *Antennipatus montceauensis* gen. nov., sp. nov., in the VAXML interchange format. The file can be downloaded from Zenodo data archive (10.5281/zenodo.50828). This can be opened with the freely available software SPIERS (spiers‐software.org).
**Video S1.** A recording showing the hand specimens of *Antennipatus montceauensis* gen. nov., sp. nov., as well as static low lighting renders, and animated multicolor lighting rigs, to emphasize the surface relief of the specimens.
